# Hypoxia reduces TGFβ1-induced corneal keratocyte myofibroblast transformation

**Published:** 2009-09-11

**Authors:** Dongmei Xing, Joseph A. Bonanno

**Affiliations:** School of Optometry, Indiana University, Bloomington, IN

## Abstract

**Purpose:**

The purpose of this study was to determine whether transient hypoxia had an effect on transforming growth factor β1 (TGFβ1)-induced rabbit corneal keratocyte myofibroblast transformation.

**Methods:**

Primary isolated rabbit corneal keratocytes were cultured in a serum-free medium. The effect of transient hypoxia treatment (1% oxygen, 4 h/day) on TGFβ1 (5 ng/ml)-induced α-smooth muscle actin (α-SM actin) expression was examined by immunofluorescence, flow cytometry, and immunocytochemistry 72 h after treatment. We found that hypoxia treatment significantly reduced the myofibroblast phenotype and α-SM actin expression that was induced by TGFβ1. To explore the possible mechanism for this effect, we screened for the effects of hypoxia on several early TGFβ-dependent signaling events including activated pSmad3, CREB (cAMP response element binding) binding protein (CBP), MAPKs (Mitogen-activated protein kinase), and RhoA by co-immunoprecipitation and western blotting.

**Results:**

Hypoxia alone increased α-SM actin expression and the association of pSmad3 to CBP, but it did not induce the myofibroblast phenotype. The levels of pERK (the extracellular signal-regulated protein kinase) and pSmad3 or the extent of the interaction between pSmad3 and CBP induced by TGFβ1 were not affected by hypoxia whereas the activation of RhoA induced by TGFβ1 was significantly reduced.

**Conclusions:**

We conclude that hypoxia can inhibit TGFβ1-induced corneal myofibroblast transformation and α-SM actin expression. Our data show that this inhibition does not occur by altering Smads or MAPK signaling but possibly by reducing the early activation of RhoA.

## Introduction

Wound healing is a complex process that includes apoptosis, cell activation and proliferation, differentiation, and myofibroblast transformation. In many tissues, wound healing is initially accompanied by ischemia/hypoxia due to the disruption of the blood supply. Hypoxia can modulate wound healing by inhibiting cytokine-induced apoptosis following wounding in cardiac myocytes [1], HepG2 (Human hepatocellular liver carcinoma cell line) cells [2], and corneal fibroblasts [3]. Hypoxia can also reduce proliferation of human dermal fibroblasts [4] following wounding. However, the direct effect of hypoxia on differentiation to the myofibroblast phenotype has not been studied.

Transforming growth factor β (TGFβ) is the major mediator responsible for myofibroblast transformation. Myofibroblasts have a contractile phenotype, which is distinguished by the expression of α-smooth muscle actin (α-SM actin), the assembly of stress fibers and focal adhesions, and the altered extracellular matrix (ECM) production leading to fibrotic wound healing in the heart [5], lung [6], and cornea [7]. TGFβ signaling is initiated by ligand binding to transmembrane receptors I/II that induce phosphorylation of Smad2/3, which combines with common Smad4, translocates to the nucleus, and recruits the coactivator, CBP (CREB binding protein)/p300, to regulate downstream genes. TGFβ also signals through the MAPK [8] or RhoA pathways [9] either independently or as modulators of Smads.

Hypoxia increases fibronectin, collagen I, and collagen IV protein expression in placental fibroblasts even though TGFβ production was not increased by hypoxia, suggesting that ECM production can be stimulated by hypoxia independent of TGFβ [10]. On the other hand, hypoxia increases TGFβ expression in HUVEC (Human Umbilical Vein Endothelial cell line) cells, which suggests an interaction of hypoxia on TGFβ bioactivity [11]. Hypoxia increases the presence of the transcriptional factor, HIF-1 α (Hypoxia inducible factor-1 alpha), which requires the coactivator, CBP/p300. This might result in a possible competition for CBP with Smads, thereby altering the TGFβ response. Indeed, competitive inhibition has been observed in cardiac fibroblasts where cAMP (cyclic Adenosine Monophosphate)-elevating agents repress TGFβ signaling by activating CREB, which recruits CBP1, effectively competing with Smad transcriptional complexes [12]. These studies suggest that hypoxia could modulate TGFβ-induced signaling during myofibroblast transformation. Whether hypoxia is pro-fibrotic or anti-fibrotic is probably dependent on the specific cell type. In corneal stromal cells, the interaction between hypoxia and TGFβ and the effect of hypoxia on TGFβ-induced myofibroblast transformation have not been studied. In a previous study, we showed that hypoxia induced a significant increase of HIF1α after 4 h of hypoxia treatment in corneal stromal cells [3]. Given the potential interactions between HIF1α and Smad signaling, we now ask if intermittent hypoxia can modulate myofibroblast differentiation and the possible involvement of Smads, MAPK, and RhoA signaling pathways.

## Methods

### Materials

The medium, additives, Alexa Fluor 488 goat anti-mouse IgG, and collagenase were purchased from Invitrogen (Cat: A-11001; Carlsbad, CA). TGFβ1 was obtained from Biosource (Camarillo, CA). Monoclonal anti-α-SM actin antibody was obtained from Sigma Chemical Company (Cat: 2547; St. Louis, MO). Phospho-MAPK family antibodies were purchased from Cell Signaling Technology (Danvers, MA).

### Cell culture and cell treatment

New Zealand White rabbit eyes were delivered from Pel-Frez (Rogers, AR). Rabbit corneal keratocytes were cultured as previously described [13]. Briefly, the epithelium was scraped off, the cornea was dissected, and the endothelium was wiped off. The entire stroma was put into DMEM (Dulbecco’s Modified Eagle’s Medium) with 2 mg/ml collagenase, 0.5 mg/ml hyaluronidase, and antibiotics at 37 °C overnight. The resultant isolated cells were washed once in DMEM and seeded in serum-free DMEM with non-essential amino acids, MEM vitamins, and sodium pyruvate onto 12 mm glass coverslips double coated with poly-lysine and collagen for immunocytochemistry and onto 60 mm or 100 mm Petri dishes for immunochemistry, pull down assays, and flow cytometry at 5×10^4^ cells/cm^2^. For immunochemistry, 5 ng/ml TGFβ1 was used to treat the cells unless otherwise stated. For 4 h and 24 h treatments, drugs were added once. For 72 h treatments, drugs were added every day with medium change every other day.

### Hypoxia treatment

For hypoxia treatment, cells were placed in a hypoxia chamber (Coy Lab Products Inc., Grass Lake, MI) equilibrated with 1% oxygen/5% CO_2_-balance nitrogen and incubated for 4 h. For phenotypic experiments, cells were treated with hypoxia (1% oxygen for 4 h every day) for three days concurrent with or without TGFβ1. For evaluation of pSmad3 and MAPK signaling, primary isolated rabbit corneal keratocytes were treated with hypoxia (1% O_2_) for 4 h with or without TGFβ1. Cells were immediately prepared for analysis after treatment.

### Immunocytochemistry

Cells were washed with fresh medium and fixed in PBS containing 3% paraformaldehyde for 10 min at room temperature. Fixed cells were then permeabilized with acetone (−20 °C for 5 min). Coverslips were dried at room temperature (RT), immediately rehydrated with PBS for 5 min, blocked in PBS:goat serum (1:1) for 1 h, incubated with anti-α-SM actin (Sigma) at 1:100 in blocking medium for 1 h at RT, and washed twice with PBS and then blocking medium for 10 min. Cells were then incubated in goat anti-mouse IgG Alexa Fluor 488 at 1:20 for 40 min (Molecular Probes, Eugene, OR), washed twice with PBS for 10 min, quickly washed with dH_2_O, and mounted in Prolong mounting medium (Molecular Probes) with DAPI (4'-6-Diamidino-2-phenylindole).

For western blots, cells were incubated with lysis buffer (Pierce, Rockford, ILf) for 5 min, scraped, and centrifuged at 14,000× g for 10 min. The supernatants were collected and stored at −80 °C. Protein concentrations were determined by the BCA method. Samples were separated on 10% polyacrylamide-SDS gels and electroblotted onto PVDF (Polyvinylidene Fluoride) membranes. After blocking with PBST/5% skim milk, the membrane was incubated overnight at 4 °C with primary antibodies against α-SM actin at 1:1,000 followed by peroxidase conjugated anti-mouse IgG at 1:1,000 for 1 h at room temperature. Signals were detected by ECL (enhanced luminol-based chemiluminescent). Each experiment was repeated at least three times. For statistical analysis, band density was analyzed using Un-scan-it gel analysis software (Silk Scientific, Orem, UT). The intensity of the band of interest is divided by the intensity of the internal control for all statistical analysis.

### Flow cytometry

Rabbit corneal keratocytes (1.5×10^6^) in 100 mm Petri dishes were detached with 0.25 mg/ml trypsin for 3 minutes, collected, and fixed in 2% paraformaldehyde for 5 min at RT, and permeabilized in 0.02% Triton with buffered PBS. Cells were then blocked with 1:1 PBS:filtered goat serum for 1 h, stained with mouse anti-αSMA antibody (1:100) in blocking medium for 40 min at RT, washed twice with PBS, and stained with goat anti-mouse IgG Alexa Fluor 488 (Invitrogen-Molecular Probes) at 1:20 for 40 min. Cells were washed twice, and at least 5,000 cells per sample were loaded in a FACSCalibur flow cytometer (Becton Dickinson, San Jose, CA) and analyzed by CellQuest software (BD biosciences PharMingen, San Diego, CA) using manual gating according to intensity of fluorescence and cell size.

### Co-immunoprecipitation

Co-immunoprecipitation (Co-IP) was performed on nuclear extracts using the ExactaCruz C: sc-45040 (Santa Cruz Biotechnology, Santa Cruz, CA) and One-Step IP-Western Kit (GenScript Corporation, Piscataway, NJ). Keratocytes (1.2×10^6^) were plated onto 100 mm plates in serum free DMEM as stated above. Monoclonal mouse anti-CBP from two companies were pooled together to increase the binding efficiency. One hundred microliters of the IP matrix with 15 μl of anti-CBP from Chemicon (Cat: MAB1133; Temecula, CA) and 15 μl of anti-CBP from R&D Systems (Cat: MAB2627; Minneapolis, MN) were incubated by end-over-end rotation overnight at 4 °C to make the IP antibody-IP matrix solution. Cells were then treated with forskolin alone or together with TGFβ1 for 4 h. Nuclear extracts were collected using the specified lysis buffer together with proteinase and phosphatase inhibitors according to the manufacturer’s protocol (Pierce). The lysate was incubated with the IP antibody-matrix by end-over-end rotation for 2 h at room temperature to pull-down CBP. Elutes from the pull-down were run on 10% polyacrylamide-SDS gels and electroblotted onto nitrocellulose membranes (Bio-Rad Laboratories, Hercules, CA). The membrane was cut in half along the 100 kDa marker. The higher molecular weight half was blotted with pooled anti-CBP antibody to check the loading. The lower molecular weight half was blotted with anti-pSmad3 (Cat: AB3226; R&D system) using GenScript One-Step Western Blot detection.

### RhoA activity assay

RhoA activity was measured semi-quantitatively by pull-down assay (Cat. BK036; Cytoskeleton, Denver, CO) according to the manufacturer’s protocols. For pull-down assays, cells in 60 mm Petri dishes were lysed in 550 μl of lysis buffer and centrifuged at 10,000× g for 2 min, and 450 μl of the supernatant was added to 25 μg Rhotekin-RBD beads and incubated for 1 h at 4 °C. Samples were washed once with washing buffer, boiled with 5× Laemmli sample buffer, run on 12% polyacrylamide-SDS gels, electroblotted onto PVDF membranes, and detected with 1:2,000 mouse anti-RhoA antibody. Thirty-five microliters of supernatant was used to detect total RhoA.

### Myofibroblast cell counting

For keratocytes cultured on 12 mm coverslips, five random distinct 200× microscopic fields were photographed on each coverslip. DAPI (+) cells were counted to obtain the total cell count. DAPI (+) and α-SM actin (+) cells were counted as myofibroblasts. Each experimental condition had duplicate or triplicate coverslips. Data was collected from about 750 cells for each condition in each experiment. Experiments were repeated at least three times giving a total of at least 2,000 cells counted per condition. Data are represented as mean±standard error.

### Statistical analysis

Data are presented as the mean±SEM for at least three separate experiments. One way analysis of variance (ANOVA) was employed for statistical analysis with significant differences determined as p<0.05.

## Results

### Hypoxia suppresses TGFβ1-induced myofibroblast differentiation

**Figure 1:**
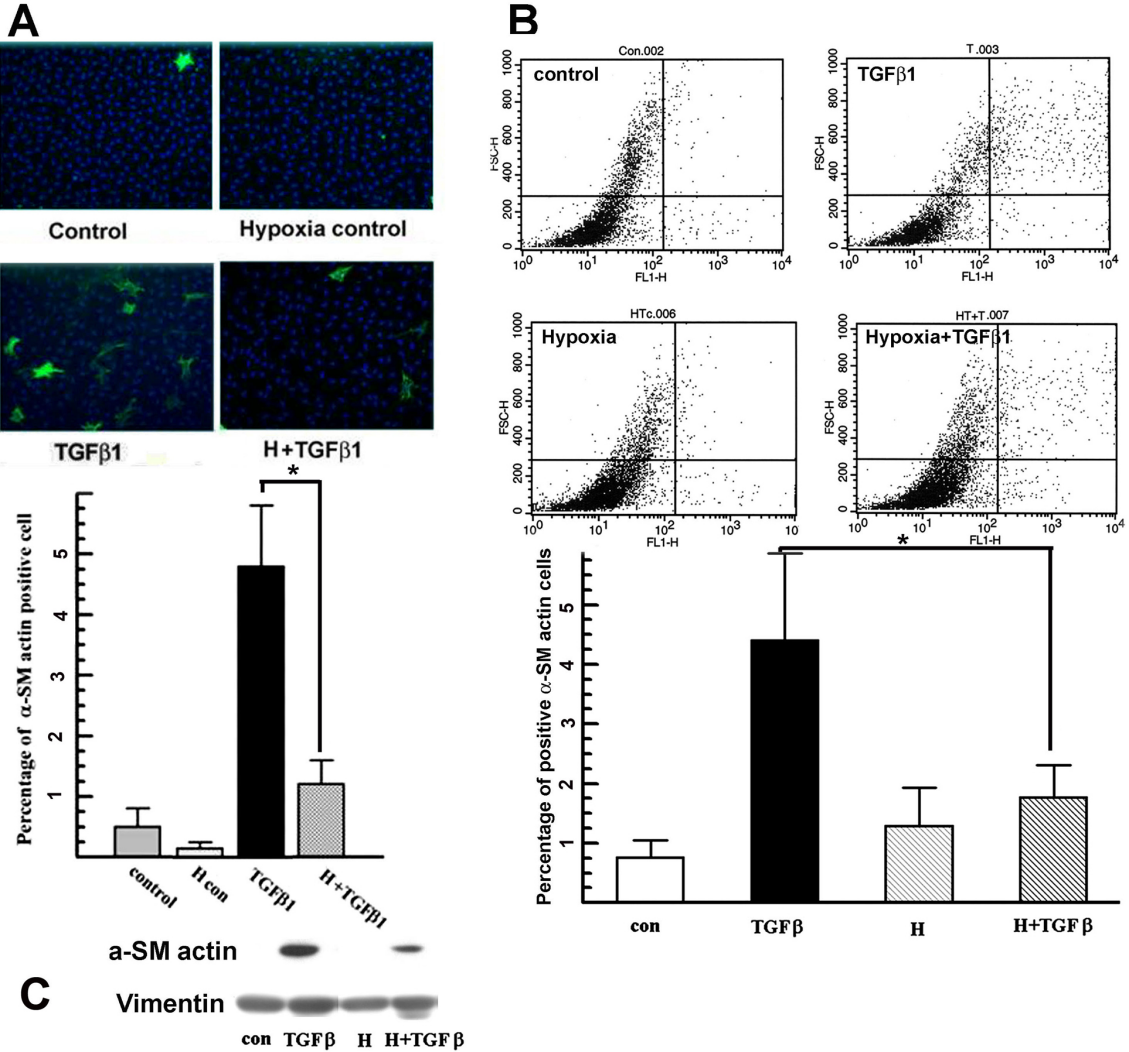
Hypoxia suppresses TGFβ-induced myofibroblast transformation. **A**: Primary cultured rabbit corneal keratocytes on coverslips were treated with hypoxia for 4 h each day for 72 h with or without TGFβ1. After treatment, cells were stained for α-SM actin (green) and nuclei (DAPI, blue). Microscope images are representatives of indicated groups (magnification: 200×). Five randomly selected fields were taken from each coverslip. The experiment was repeated three times. The bar graph shows the percentage of α-SM actin positive cells over total cell count in each group from immunofluorescence analysis. Error bars represent the standard error of the mean (n=3 experiments). The asterisk indicates that the indicated groups were significantly different from TGFβ (p<0.05). **B**: Primary rabbit keratocytes on 100 mm Petri dishes were treated the same as in (**A**), stained for α-SM actin and then analyzed by flow cytometry. Representative images show α-SM actin fluorescence on the x-axis and forward scatter on the y-axis. Bar graph shows the percentage of α-SM actin positive cells over total cell count in each group. The experiment was repeated three times. Error bars represent the standard error of the mean (n=3). The asterisk denotes that the indicated groups were significantly different from TGFβ (p<0.05). **C**: Primary rabbit keratocytes on the Petri dish were treated the same as in (**A**). Whole cell lysates were collected immediately after treatment and analyzed by western blot for α-SM actin. The image shown is the representative western blot of three experiments.

We used periodic hypoxia (only 4 h/day), which we know induces HIF1α [3], to test the effect of hypoxia on rabbit keratocyte myofibroblast transformation. Figure 1A shows that hypoxia treatment significantly reduced the TGFβ1-induced myofibroblast phenotype from 4.8%±1% to 1.2%±0.3% of the cell populations as determined from immunocytochemistry. Hypoxia alone (no TGFβ) over three days did not increase the proportion of myofibroblasts compared to the control (Figure 1A). To confirm that hypoxia significantly reduced the proportion of TGFβ1-induced myofibroblasts, we repeated the experiment using quantitative flow cytometry analysis for α-SM actin. Figure 1B shows that hypoxia treatment significantly reduced TGFβ1-induced myofibroblast differentiation from 4.0%±1.1% to 1.8%±0.4% 72 h after treatment. Overall, Figure 1 shows that hypoxia reduces TGFβ1-induced myofibroblast transformation of corneal keratocytes and that hypoxia alone does not induce the myofibroblast phenotype. These results were confirmed at the protein level by western blotting. Figure 1C shows that TGFβ1 induces a significant amount of α-SM actin expression while hypoxia suppresses TGFβ1-dependent induction of α-SM actin expression.

### Hypoxia does not reduce TGFβ1-induced pSmad3 level

To explore the mechanism by which hypoxia suppresses myofibroblast transformation, we examined the effect of hypoxia on early TGFβ-induced signaling events. Smad signaling is the major TGFβ-induced signaling pathway. pSmad3 is needed for TGFβ-induced α-SM actin expression in fibroblasts [14]. pSmad3 is a transcriptional factor that is upregulated within 4 h of TGFβ1 treatment in corneal keratocytes [15]. Therefore, we examined the effect of hypoxia on TGFβ1-induced α-SM actin expression and Smad3 phosphorylation at 4 h.

**Figure 2:**
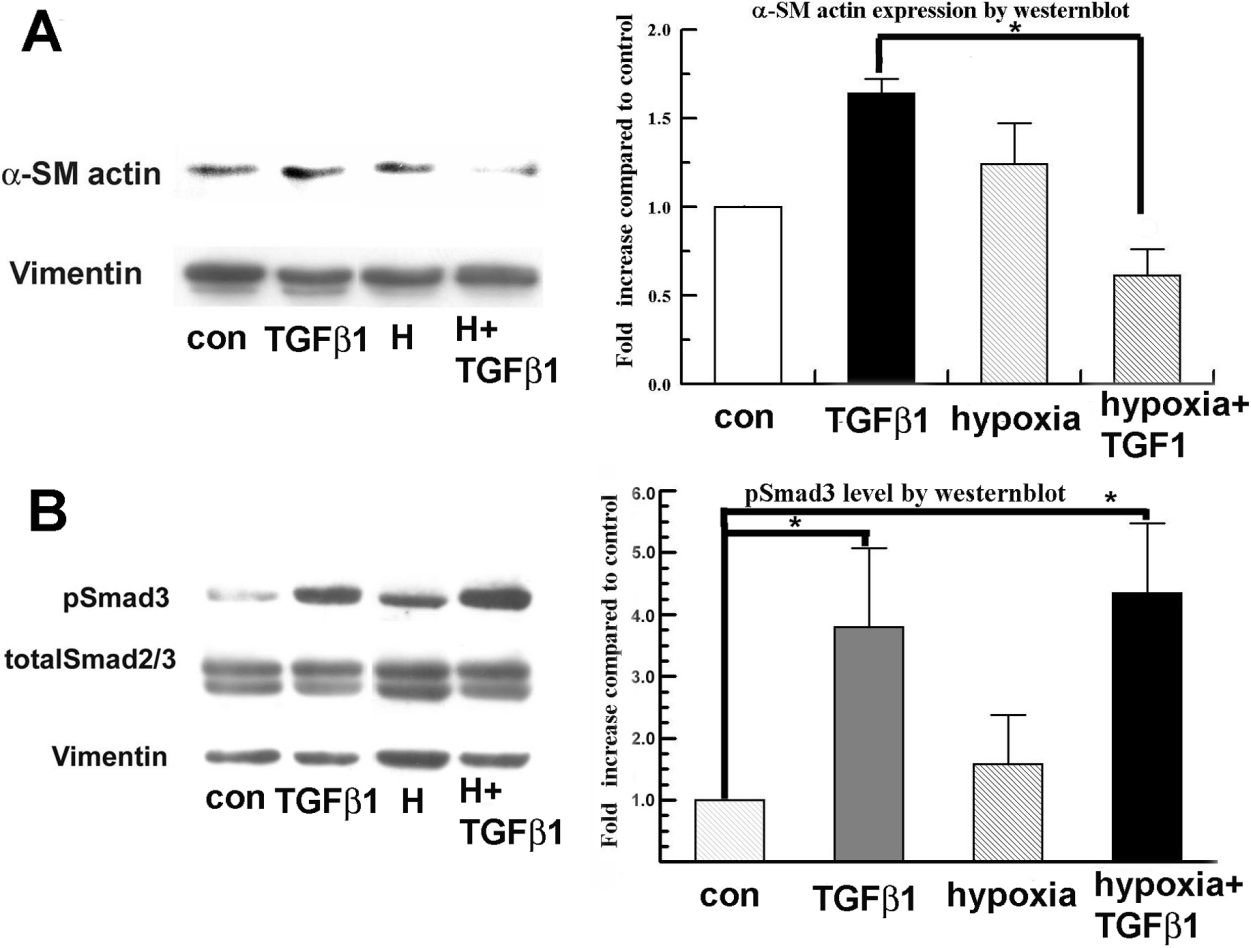
Hypoxia treatment reduces TGFβ1 induced α-SM actin but not Smad3 phosphorylation. Primary isolated rabbit keratocytes were treated with hypoxia together with or without TGFβ1 for 4 h. Whole cell lysates were collected immediately after treatment and analyzed by western blot for α-SM actin (**A**), pSmad3 (**B**), and totalSmad2/3. Vimentin was used as a loading control. Bar graphs show fold increase of band intensity of each group compared to vimentin (**A**) and to total Smad2/3 (**B**). Error bars represent the standard error of the mean (n=3). The asterisk denotes p<0.05.

Figure 2A shows that TGFβ1 increases α-SM actin expression by 1.7±0.1 fold after only 4 h. Interestingly, hypoxia alone revealed a slight but not significant increase (1.3±0.2 fold increase) in α-SM actin expression over the control. Hypoxia significantly reduced the α-SM actin expression induced by TGFβ1 at 4 h, which is consistent with the reduction in the myofibroblast phenotype (Figure 1). Figure 2B shows that TGFβ1 induced a significant increase (3.75±1.25 fold increase) in pSmad3 4 h after treatment in rabbit keratocytes compared to the control. Hypoxia by itself slightly increased pSmad3, but this increase was not significant. The combination of hypoxia with TGFβ1 had no effect on induced pSmad3 levels, indicating that inhibition of TGFβ-induced α-SM actin expression by hypoxia is not through interference with pSmad3.

### Hypoxia does not alter the level of interaction of CBP and pSmad3 induced by TGFβ1

TGFβ1-induced Smad signaling needs the binding of the coactivator, CBP/p300, to exert its function. The transcription factor, HIF1α, which is induced by hypoxia, also needs CBP as a coactivator. Concurrent activation of Smads and Hif1α might lead to a competitive inhibition of pSmad3-CBP interaction. We tested this possibility by co-immunoprecipitation of CBP from nuclear extracts 4 h after treatment and then probed for pSmad3.

**Figure 3:**
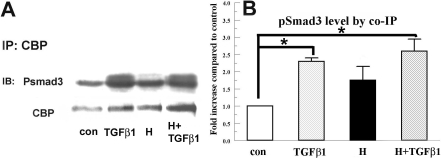
Hypoxia does not reduce TGFβ1 induced interaction between pSmads and CBP in rabbit keratocytes. Nuclear extracts were collected 4 h after corresponding treatment and immunoprecipitated with anti-CBP antibody. Eluates were separated by SDS–PAGE and probed for pSmad3. Blots were also probed for CBP as an internal control. **A**: Image shows a representative western blot. **B**: Bar graph shows the relative change of pSmad3 over the control group. Error bars represent the standard error of the mean (n=3). The asterisk denotes that the indicated groups were significantly different from control (p<0.05).

Figure 3 shows that hypoxia or TGFβ1 alone induced a significant increase in interaction between pSmad3 and CBP (1.8±0.4 and 2.4±0.1 fold increase, respectively). However, hypoxia+TGFβ1 (2.6±0.3 fold increase) did not significantly change the TGFβ-induced pSmad3-CBP interaction. These results indicate that the reduction of TGFβ-induced myofibroblast formation and α-SM actin expression by hypoxia is not through the reduction of pSmad3 or pSmad3-CBP interaction.

### TGFβ induced pERK is not altered by hypoxia

**Figure 4:**
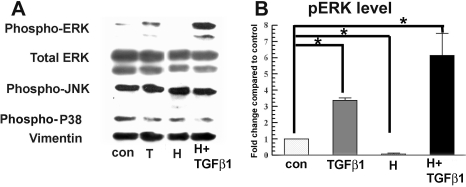
Hypoxia inhibition of TGFβ1-induced α-SM actin is independent of MAPK activation. **A**: Whole cell lysates were collected 4 h after treatment and assayed by western blot for pERK, total ERK, pJNK, and phospho-p38 as indicated. Vimentin was used as a loading control. **B**: The bar graph shows the relative increase of pERK in each group over the control. Error bars represent standard error of the mean (n=3). The asterisk indicates that the indicated groups were significantly different from control (p<0.05).

In addition to increasing pSmad3 activation, TGFβ significantly increases phosphorylation of ERK at 4 h. Both TGFβ-induced αSM actin and pERK are significantly reduced by the MEK (mitogen-activated protein kinase kinase) inhibitor, U0126, indicating an important role for pERK in inducing myofibroblasts [15]. Figure 4 shows that hypoxia alone significantly reduced pERK below the control level in rabbit keratocytes. Interestingly, hypoxia did not reduce TGFβ-induced ERK phosphorylation. Instead, pERK was significantly increased relative to TGFβ alone. Figure 4 also shows that there was no change in total ERK, pJNK (phosphorylated jun kinase), or phospho-p38 levels in all experimental groups at 4 h after treatment. These results indicate that hypoxia inhibition of TGFβ1-induced α-SM actin expression in rabbit keratocytes is not through interference with ERK activation.

### Hypoxia reduces TGFβ-induced active RhoA

**Figure 5:**
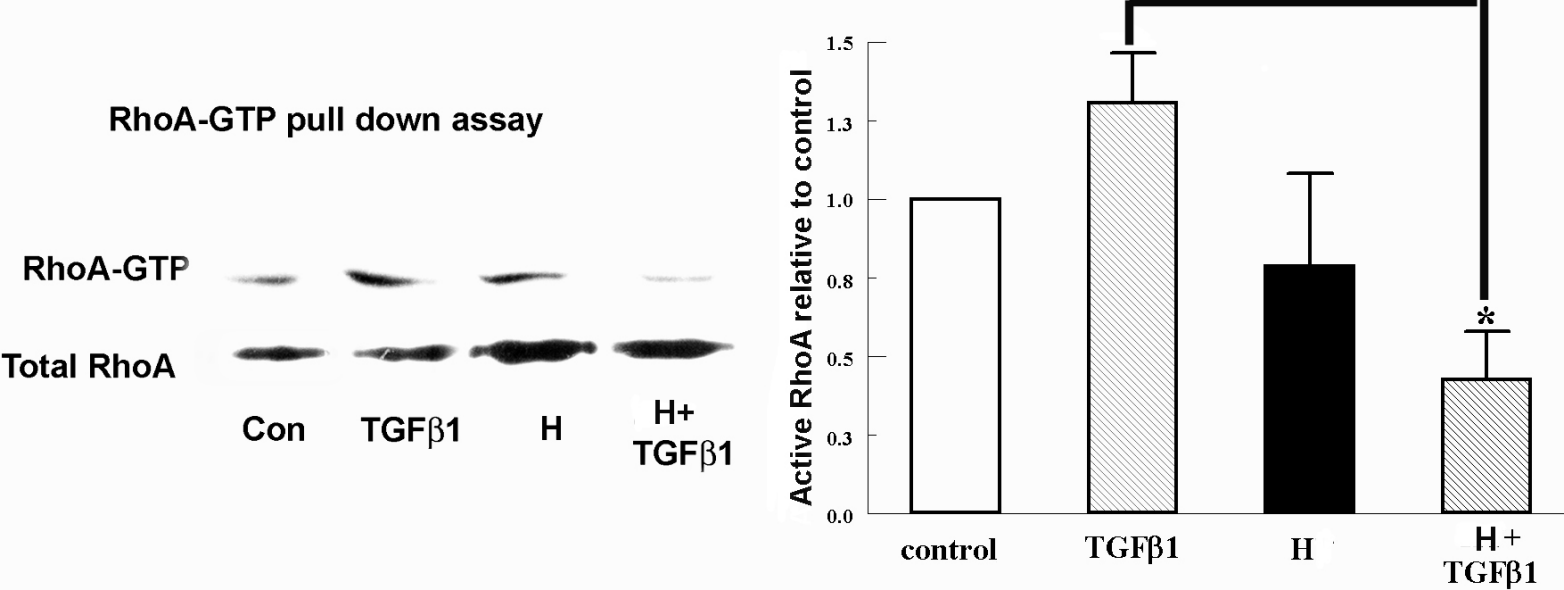
RhoA activation is involved in hypoxia inhibition. Cells were treated under normoxia (control) or hypoxia (H) with or without 5 ng/ml TGFβ1 for 7 min. GTP bound RhoA was pulled down and assayed by western blot with anti-RhoA antibody. Total RhoA from whole cell lysates was detected as the relative control. The representative image of three experiments is shown here. Error bars represent standard error of the mean (n=3). The asterisk denotes that the indicated groups were significantly different from TGFβ1 (p<0.05).

TGFβ can also activate the small GTP-binding protein, RhoA. The RhoA pathway has a role in α-SM actin expression in rabbit corneal myofibroblasts [16]. Recently, we found that in rabbit keratocytes, TGFβ1 transiently induces GTP-RhoA between 5 and 15 min, peaking at approximately 7 min, and that interfering with RhoA activation by ROCK (Rho-associated coil-containing protein kinase) inhibition reduced αSM actin expression after 4 h of TGFβ exposure [15]. Here, we tested the effect of hypoxia on GTP-RhoA following 7 min of TGFβ1 treatment. Cells were pre-equilibrated in hypoxic media for 4 h. TGFβ was added to the cells within the hypoxia chamber, and cells harvested for western blot analysis 7 min later. Figure 5 shows that TGFβ1 significantly increased the GTP-RhoA level (1.38±0.15 fold increase) after 7 min compared to the control. Hypoxia alone did not significantly change GTP-RhoA. However, TGFβ+hypoxia significantly reduced the GTP-RhoA level to less than the control level.

## Discussion

In this study, we report for the first time that hypoxia reduces TGFβ1-induced myofibroblast transformation in primary isolated rabbit keratocytes. Our results demonstrate that hypoxia inhibits TGFβ1-induced keratocyte from myofibroblast transformation (Figure 1) and also reduces α-SM actin expression at 4 h (Figure 2A) and 72 h (Figure 1B)  after TGFβ1 treatment.

Hypoxia as an individual stress factor can induce α-SM actin expression in fetal cardiac fibroblasts [17]. This appeared to happen with rabbit keratocytes (Figure 2A), but this induction was not significant. On the other hand, hypoxia did not induce α-SM actin-containing filaments at 72 h (Figure 1A,C). This may be due to the lower α-SM actin level induced by hypoxia relative to TGFβ and the altered expression of associated cytoskeletal proteins needed for the alignment of stress fibers [18].

To examine the possible mechanism of hypoxia’s effect on myofibroblast transformation, we explored early events in the signaling pathways induced by TGFβ. Smads are the major effectors of TGFβ signaling. pSmad3 recruits the transcriptional coactivator, CBP/p300, for DNA binding [19]. Recent studies showed that one mechanism of inhibition of TGFβ-induced myofibroblast transformation and collagen synthesis is by increased competition for CBP/P300 with Smad signaling [12]. Figure 2 showed that pSmad3 is activated by TGFβ within 4 h, and α-SM actin expression is significantly increased. This increase in α-SM actin is suppressed by concomitant hypoxia. We found that hypoxia treatment alone may induce Smad signaling by increasing the interaction of pSmad3 with CBP in rabbit keratocytes (Figure 3), but this induction was not statistically significant. However, hypoxia and TGFβ together did not reduce the level of pSmad3 (Figure 2) or pSmad3/CBP interaction relative to TGFβ alone (Figure 3). Overall, modulation of Smad signaling is not likely to be involved in the hypoxia inhibition of myofibroblast transformation.

We showed that TGFβ upregulates pERK in rabbit keratocytes, which is consistent with previous findings in lung fibroblasts where pERK upregulation contributes to myofibroblast formation [20]. The reduction of ERK phosphorylation has been shown to be one mechanism that interferes with TGFβ signaling in cardiac fibroblasts [12]. Furthermore, inhibition of ERK phosphorylation in keratocytes significantly reduced TGFβ-induced α-SM actin expression [15]. Here, we found that hypoxia treatment alone decreases pERK significantly in rabbit keratocytes. This regulation of pERK by hypoxia is cell type specific since hypoxia has been shown to be a strong inducer for ERK phosphorylation in other cell types [21]. Interestingly, our experiments showed that even though hypoxia alone reduces pERK, it significantly increases ERK phosphorylation induced by TGFβ1 (Figure 4). To our knowledge, this paradoxical effect on ERK phosphorylation by hypoxia with/without TGFβ has not been reported. This result could be consistent with induction of dissimilar signaling components by hypoxia and TGFβ1, which together lead to an enhanced ERK phosphorylation. Whatever the mechanism of this effect on pERK, the hypoxia inhibition of myofibroblast differentiation is not through reduction of pERK.

RhoA activation is involved in TGFβ1-induced α-SM actin expression in several cell types including corneal fibroblasts and keratocytes [13,22,23]. In corneal myofibroblasts, TGFβ1-induced α-SM actin expression involves RhoA/ROCK and RhoA/mDia1 pathways and is suppressed by a ROCK inhibitor [16]. Recently, we found that in rabbit keratocytes, TGFβ1 transiently induces GTP-RhoA between 5 and 15 min, peaking at approximately 7 min and that interfering with RhoA activation by ROCK inhibition reduced α-SM actin expression after 4 h of TGFβ exposure [15]. Hypoxia has been shown to activate RhoA signaling in pulmonary arterial smooth muscle cells and A549 cells [24,25]. In rabbit keratocytes, we did not find a significant change in RhoA activation by hypoxia. But in the presence of hypoxia, the initial TGFβ1-induced activation of RhoA in corneal keratocytes was significantly reduced (Figure 5), suggesting that the RhoA pathway might be involved in hypoxia inhibition of TGFβ1-induced α-SM actin. Further studies are needed to test the hypothesis that RhoA is responsible for the hypoxia-dependent inhibition of myofibroblast formation.

In summary, hypoxia reduces TGFβ1-induced myofibroblast transformation in rabbit keratocytes in vitro. Hypoxia does not interfere with TGFβ-induced changes in pSmad3, CBP interaction, or pERK. However, hypoxia reduces RhoA activation induced by TGFβ. Hypoxia alone induces α-SM actin expression, consistent with enhanced Smad signaling, but does not induce the myofibroblast phenotype. In vivo, corneal hypoxia alone, for example that produced by wearing contact lenses, also does not induce the myofibroblast phenotype. This suggests that intermittent hypoxia, for example that achieved by contact lens wear, could be investigated as a possible tool to reduce fibrosis following corneal wounding or surgery.
